# Relationship between alcohol and primary headaches: a systematic review and meta-analysis

**DOI:** 10.1186/s10194-023-01653-7

**Published:** 2023-08-23

**Authors:** Bartłomiej Błaszczyk, Marcin Straburzyński, Mieszko Więckiewicz, Sławomir Budrewicz, Piotr Niemiec, Martyna Staszkiewicz, Marta Waliszewska-Prosół

**Affiliations:** 1 Faculty of Medicine, Wroclaw Medical University, Wroclaw, Poland; 2 Department of Family Medicine and Infectious Diseases, University of Warmia and Mazury, Olsztyn, Poland; 3 Department of Experimental Dentistry, Wroclaw Medical University, Wroclaw, Poland; 4 Department of Neurology, Wroclaw Medical University, Borowska 213 Str, 50-556 Wroclaw, Poland

**Keywords:** Migraine, Tension-type headache, TTH, Cluster headache, Drinking, Pain, Alcohol

## Abstract

**Background:**

Headache is one of the most common neurological symptoms. Many previous studies have indicated a relationship between primary headaches and alcohol. Drinking has been associated with increased risk of tension-type headache (TTH) and migraine. However, recently published studies have not confirmed this relationship. The existing literature is inconclusive; however, migraine patients avoid alcohol. Therefore, the primary objective was to provide a reliable assessment of alcohol intake in people with primary headaches; the secondary objective was to identify any potential relationship between alcohol consumption and headache risk.

**Methods:**

This study was based on PubMed, Embase and Web of Science database searches performed on 11 July 2023. This systematic review was registered in PROSPERO (CRD42023412926). Risk of bias for the included studies was assessed using the Joanna Briggs Institute critical appraisal tools. Meta-analyses were performed using Statistica software. The Risk Ratio (RR) was adopted as the measure of the final effect. Analyses were based on a dichotomous division of the respondents into "non-drinkers" and "drinkers" for headache patients and matched non-headache groups.

**Results:**

From a total of 1892 articles, 22 were included in the meta-analysis. The majority demonstrated a moderate or high risk of bias. The first part of the meta-analysis was performed on data obtained from 19 migraine studies with 126 173 participants. The risk of migraine in alcohol drinkers is approximately 1.5 times lower than in the group of non-drinkers (RR = 0.71; 95% CI: 0.57–0.89). The second part involved 9 TTH studies with 28 715 participants. No relationship was found between TTH diagnosis and alcohol consumption (RR = 1.09; 95% CI: 0.93–1.27). Two of the included cluster-headache articles had inconclusive results.

**Conclusions:**

Alcohol consumption and migraine are inversely correlated. The exact mechanism behind this observation may indicate that migraine leads to alcohol-avoidance, rather than alcohol having any protective role against migraine. There was no relationship between TTH and drinking. However, further studies related to primary headaches and alcohol consumption with low risk of bias are required. Additionally, patients and physicians should consider the latest medical data, in order to avoid the myths about alcohol consumption and primary headaches.

## Introduction

Headaches are one of the most common neurological symptoms related to the sensation of pain [1] and cause a decrease in patients’ quality of life [2]. Their global prevalence is estimated at 52% of the population [3]. Headache disorders are classified according to the third edition of the International Classification of Headache Disorders (ICHD) [4, 5] as either primary headaches, secondary headaches or neuropathies and facial pains [4, 6].

The most prevalent primary headache disorder is tension-type headache (TTH) with a prevalence of 40%, followed by migraine (> 10%); while cluster headache (CH) occurs in only 0.12% of the general population [7, 8]. Furthermore, TTH is the most common neurological disorder in the world [9] and presents significantly more frequently in women of all ages, races and socioeconomic status than in men [10–12]. The peak TTH incidence occurs in people 30–39 years old. The symptoms include bilateral, pressing or tightening pain in the forehead, occiput or neck regions [13, 14]. Migraine is most often diagnosed between the 25th and 55th year of life, especially in women [5, 15]. Migraine attacks last 4–72 h and are characterized by a unilateral, throbbing headache with vomiting, nausea and photophobia or are preceded by aura. CH is considered a rare disorder and commonly affects men aged 20–30 years [16]. Its attacks appear 1–8 times a day during the active phase. These headaches are severe, located around the orbit with cranial autonomic symptoms including tearing, miosis, ptosis and anxiety [17].

Alcohol is a psychoactive substance that leads to many health problems such as cancers and traffic accidents; it directly causes impairment in attention, cognition and dexterity, and aggressiveness and loss of control [18–20]. In the USA, 51% of adults consumed alcohol in the last year; additionally, 11% of those over 50 years old and 6% over 65 age reported the symptoms of alcohol abuse or dependence [21]. In Europe, 60% of adults over 60 years of age are current drinkers, and 20% of these had higher levels of consumption than the general population [22]. Statistically, males drink more alcohol than women and have more alcohol-related behavioral disorders [23]. Drinking problems occur in every age, but in the 25–49 age group, alcohol has the highest impact on mortality caused by cancer deaths and also life disability [24, 25].

Many previous studies have proved the relationship between primary headaches and alcohol. Alcohol consumption is associated with increased risk of TTH and migraine [26–30], or as a trigger of headache attacks [31–33]; indeed, there may be increased mortality in patients with migraine [32, 34]. However, recently published studies have not confirmed the relationship between alcohol and headaches [34–36]. Data related to this area is inconclusive; however, migraine patients avoid alcohol drinking [37, 38] unlike their young peers, who often drink alcohol to have fun, cope with problems, relax and maintain friendships [39–41]. Therefore, in order to provide the most objective clues for a normal lifestyle among headache patients, based on existing single studies, our systematic review collates the data about alcohol consumption and primary headaches. The primary objective was to reliably and objectively assess alcohol intake in people with primary headaches, and the secondary objective was to identify any potential relationship between alcohol consumption and headache risk.

## Methods

The systematic review presented in this paper was conducted following the Preferred Reporting Items for Systematic Reviews and Meta-Analyses 2020 (PRISMA 2020) guidelines [42]. The systematic review was registered on PROSPERO (protocols in the International Register of Systematic Reviews) [43]—CRD42023412926.

### Data sources and search terms

In order to perform a systematic review, articles were searched in three databases: PubMed, Embase, Web of Science on July 11, 2023. There were no limitations regarding the time frame for the data search. To satisfy the aim of this paper, the below key terms were used: ("primary headache" OR "migraine" OR "tension-type headache" OR "trigeminal autonomic cephalgia" OR "cluster headache" OR "paroxysmal hemicrania" OR "short-lasting unilateral neuralgiform headache attacks" OR "SUNCT" OR "hemicrania continua" OR "primary cough headache" OR "primary exercise headache" OR "primary headache associated with sexual activity" OR "primary thunderclap headache" OR "cold-stimulus headache" OR "primary stabbing headache" OR "nummular headache" OR "hypnic headache" OR "new daily persistent headache") AND ("alcohol" OR "alcohol drinking" OR "alcoholic beverage" OR "alcoholic consumption" OR "alcohol use" OR "alcohol intake" OR "wine" OR "beer" OR "vodka" OR "gin" OR “drinking”) AND ("correlation" OR "relationship" OR "effect" OR "influence" OR "interaction" OR "trigger" OR "associated" OR "association" OR "connection" OR "factor" OR "relation" OR "impact" OR "cause" OR "induce" OR "risk factor"). In PubMed and Web of Science, the above key terms were used in all fields; in Embase, the search was performed in titles, abstracts and keywords.

### Literature search

After creating and using search terms in databases, the results were searched by three authors (BB, PN and MS^1^) independently. Then, the results were compared by researchers and duplicates were removed. Any remaining articles were screened by title or abstract randomly by the authors (BB, PN and MS^1^) with the below presented inclusion/exclusion criteria and PRISMA 2020 guidelines. Hence, papers that did not meet the inclusion criteria were excluded. In the final step, to assess the exact number of included articles, the authors (BB, PN and MS^1^) read the appropriate full-text papers and confirmed their relevance to the primary objective. In cases of conflict between authors in terms of the inclusion of a particular paper, the fourth researcher (MWP) decided upon a solution to the problem following discussion.

#### Inclusion and exclusion criteria

Studies were taken into consideration if they met the following criteria: English studies available in full-text, original papers, articles containing data about alcohol intake in patients suffering from primary headaches. Primary headaches had to be diagnosed using the appropriate criteria (IHS, ICHD). Alcohol consumption was considered in all patients, ages, populations, with all comorbidity diseases and with any primary headaches. The studies had to contain the exact information, in which way were assessed the alcohol consumption e.g. daily drinking, consumption in the last week, drinking habits during the last 2 months period etc. However, from this data, there clearly had to be an extracted division on “drinkers” and “non-drinkers”. The overall results had to be presented in a clear way with the exact numbers of drinking patients and abstainers, and these had to be assigned to the particular type of primary headaches. Additionally, results of alcohol consumption had to be compared with other groups of people who do not suffer from a particular headache (the article had to include a control/healthy population to compare data). In rare cases where a paper lacked a healthy group but where the focus was on the assessment of primary headaches, the control group was made up of another type of primary headache, whereby larger groups of patients with headache were compared to smaller groups with other headaches.

Exclusion criteria included non-English studies; non-original studies as case reports, case series, reviews, conference abstracts, book chapters; animal studies; assessments of alcohol reaction on primary headaches in the molecular pathway; primary headaches diagnosed by self-report and ICD scale, lack of presented techniques to assessments of alcohol intake habits, lack of description of alcohol intake and lack of assigned patients to a detailed type of headache and alcohol intake, lack of control/healthy group for comparison.

### Data extraction

From each included paper, three authors (BB, PN and MS^1^) extracted the following data: study authors, country where study was conducted, criteria used to diagnose headache, number of drinkers and non-drinkers in primary headache and matched control groups, type of headache, type of control group to compare data and methods for assessment of alcohol intake. This data are presented in Table 1.

**Table 1:** Features of the studies included in this paper. Abbreviations: ICHD – International Classification of Headache Disease, second and third versions; IHS – International Headache Society; TTH – tension-type headache

No	Author	Country	Criteria to diagnose headache	Type of headache	Drinkers	Non-drinkers	Overall number of headache patients	Control group	Drinkers	Non-drinkers	Overall number of control patients	Methods used to assessment drinking	Criteria to recognize habits for alcohol consumption
1.	Lisicki M et al. [44]	International	ICHD-3	Migraine	20	39	59	Non-headache	30	47	77	Self-designed questionnaire	Frequency divided on: never, ≥ once a month, ≥ once a week and every day for wine, beer and other alcoholic beverages
2.	Schramm S et al. [45]	Germany	ICHD not reported the edition	Migraine	49	367	416	Non-headache	96	367	463	Questionnaire	Alcohol was assessed as the average consumption of different beverages (beer, red wine, white wine, spirits, and cocktails) within the last 4 weeks. The proportion of pure alcohol per beverage was calculated multiply by frequency of drinking. All beverages drinked by each person were summed up and presented as the total consumption of pure alcohol g/day
3.	Aamodt AH et al. [46]	Norway	IHS from 1988	Migraine	4012	2197	6209	Non-headache	30,421	14,749	45,170	Questionnaire	Abstinence, number of times per month and the number of glasses of beer, wine or liquor during the last 2 weeks
4.	Hagen K et al. [36]	Norway	ICHD-2	Migraine	24	65	89	Non-headache	967	1086	2053	Self-designed questionnaire	The participants were divided into four groups: no use, less than four times per month, four to seven times per month, or at least eight times per month
				Tension type headache	749	52	801	Non-headache	11,680	1086	12,766		
5.	Misakian AL et al. [47]	International	Modified version of the IHS	Migraine	149	922	1071	Non-headache	1717	6839	8556	Self-designed questionnaire	Alcohol use was divided into 4 groups: never/rarely, 1–3 times/month, 1–6 times/week, daily
6.	Luo J [48]	United States of America	ICHD-3	Migraine	51	458	509	Non-headache	359	2278	2637	Questionnaire	Alcohol was divided into 2 groups: alcohol drinker (had any alcohol in the last 24 h) or non-drinker (lack of alcohol within last 24 h)
7.	Özcan RK et al. [49]	Turkey	ICHD-3 beta	Migraine	1	141	142	Non-headache	1	141	142	Questionnaire	Alcohol use was defined by identifying the quantity consumed in the last month
8.	Sarker MA et al. [50]	Bangladesh	IHS-2	Migraine	19	119	138	Non-headache	4	272	276	Questionnaire	The patients were asked about consuming any alcoholic drink in the last 2 weeks
9.	Le H et al. [51]	Denmark	IHS	Migraine	5744	2200	7944	Non-headache	19,073	4431	23,504	Questionnaire	Alcohol consumption was divided into 3 groups: never/seldom, monthly, weekly
10.	Schramm SH et al. [52]	Germany	ICHD-2	Migraine	2	722	724	Non-headache	6	1068	1074	Questionnaire	2 groups were distinguished: drinking (yes) was defined as daily or almost daily drinking of alcoholic beverages and drinking (no) was defined as no or casual drinking of alcoholic beverages
				Tension type headache	11	1544	1555		6	1023	1029		
11.	Kim BS et al. [53]	Korea	ICHD-3	Migraine	3	48	51	Non-headache	23	79	102	Questionnaire + interview	Alcohol drinking was positive if regular drinking were once a week
12.	Gür-Özmen S et al. [54]	Turkey	ICHD-2	Migraine	4	166	170	Non-headache	1	169	170	Questionnaire	The mean daily alcohol intake was calculated using the beverage-specific quantity-frequency measure: number of days with alcohol consumption (beer, wine, and spirits)mean daily alcohol consumption over the past week. Amount of wine and beer in liters and spirits in glasses was assessed and calculated in grams per day
				Tension type headache	0	170	170		1	169	170		
13.	Yoon MS et al. [55]	Germany	ICHD-2	Migraine	320	4244	4564	Non-headache	555	3086	3641	Questionnaire	Alcohol was assessed by drinking daily and not daily alcohol consumption
				Tension type headache	126	1107	1233		555	3086	3641		
14.	Kaltseis K et al. [26]	International	ICHD-3	Migraine	154	31	185	Non-headache	729	222	951	Interview	Alcohol intake was divided into 2 groups: drinkers and non-drinkers. Drinkers positively answer the question whether drinked alcohol last week and disclose the type, amount, and frequency of the consumed beverages. Then, the alcohol consumption was calculated using the formula: amount of alcohol beverage in milliliters Vol% 100 0:8 ¼ alcohol in grams:
				Tension type headache	479	78	557		729	222	951		
15.	Pellegrino Baena C et al. [56]	Brasil	IHS	Migraine	179	1060	1239	Non-headache	1096	131	1227	Questionnaire	Alcohol use was defined as never, former or present. Whereby never was assumed as non-drinkers, the rest of participants (former, present) were assumed as drinkers
16.	McMurtray AM et al. [57]	United States of America	ICHD-2	Migraine	2	22	24	Non-headache	7	7	14	Questionnaire	Alcohol use was defined as none, past or current. Whereby none was assumed as non-drinkers, the rest of participants (past, current) were assumed as drinkers
				Tension type headache	2	19	21	Non-headache	7	7	14		
17.	Lebedeva ER et al. [27]	International	ICHD-3	Migraine	129	367	496	Non-headache	282	732	1014	Interview	Alcohol intake was divided into consumption of light alcoholic beverages (at least 0.5 L per week) and strong alcoholic beverages (150 g per week)
				Tension type headache	639	1196	1835	Non-headache	282	732	1014		
18.	Schramm SH et al. [58]	German	ICHD-2	Migraine	114	1564	1678	Non-headache	555	3086	3641	Questionnaire	Alcohol was assessed by drinking daily and not daily alcohol consumption
				Tension type headache	11	1587	1598		555	3086	3641		
19.	Scher AI et al. [59]	Netherland	IHS	Migraine	304	316	620	Non-headache	3235	1900	5135	Questionnaire	Alcohol consumption was categorized as 0, < 1, 1 to 3 and + 4 drinks per day
20.	Rasmussen BK [60]	Denmark	IHS	Tension type headache	53	114	167	Non-tension type headache (migraine)	38	81	119	Interview	Alcohol intake was recorded as number of drinks (beers. glasses of wine. and glasses of strong alcohol) per week (1 drink = 10 g of alcohol)
21.	Lund N et al. [61]	Denmark	ICHD-2	Cluster headache	242	157	397	Non-headache	179	21	200	Questionnaire + interview	Alcohol intake was positive if participants had reported any intake of alcohol per week during the past year
22.	Lambru G et al. [62]	Italy	ICHD-2	Cluster headache	149	51	200	Non-cluster headache (migraine)	113	87	200	Questionnaire	Alcohol consumption, measured in alcohol units per day and if there was positive intake the division on: mild drinkers, < 4 units/day; moderate drinkers, 4–8 units/day; heavy drinkers, > 8 units/day was conducted

### Assessment of risk bias

Due to the inclusion of many study designs, the risk of bias was evaluated using tools adjusted to the type of study. The Joanna Briggs Institute (JBI) critical appraisal tools were used for cross-sectional, cohort and case control studies [63]. According to the appropriate JBI checklist, cross-sectional studies had to be conducted on the basis of eight questions, case-controls had ten questions, while cohort studies contained 11 questions. Possible answers were “Yes”, “No”, “Unclear” or “Not applicable”. If a cross-sectional study received seven or more positive answers, a case–control eight and a cohort study nine, ten or 11, their assessments were described as having a low risk of bias. A high risk of bias was reported when a cross-sectional study received five or fewer “yes” responses, a case–control fewer than six and a cohort study below seven. A moderate risk of bias was assigned when the paper received positive answers between mentioned ranges. The assessments were conducted by three researchers (BB, PN and MS^1^) separately, then the fourth author (MWP) compared this data and made a final decision.

### Statistical analysis

Meta-analyses were performed using Statistica v.13.3 software (Tibco Software Inc.). Due to the type of the available data (2 × 2 tables), the relative-risk ratio (RR) risk was adopted as the measure of the effect. Heterogeneity analyses were carried out using the Q statistic based on ✗^2^ and the corresponding *p* value. To determine the proportion of heterogeneity between the study estimates, the I^2^ statistic was used. Since the result of the heterogeneity test proved to be highly significant (*p* < 0.001), a random effect model was used for the meta-analysis.

In order to detect publication bias, the symmetry of funnel plots was analyzed using the Trim and Fill method, and the Egger test as well as the Begg and Mazumdar test were used. In order to assess the extent to which the assumptions of the meta-analysis and the studies included therein influenced the overall results, a sensitivity analysis was also performed. In all statistical tests, *p* < 0.05 was considered significant. Dichotomous division of the respondents into "non-drinkers" and "drinkers" was used.

## Results

### Study selection

**Fig. 1:**
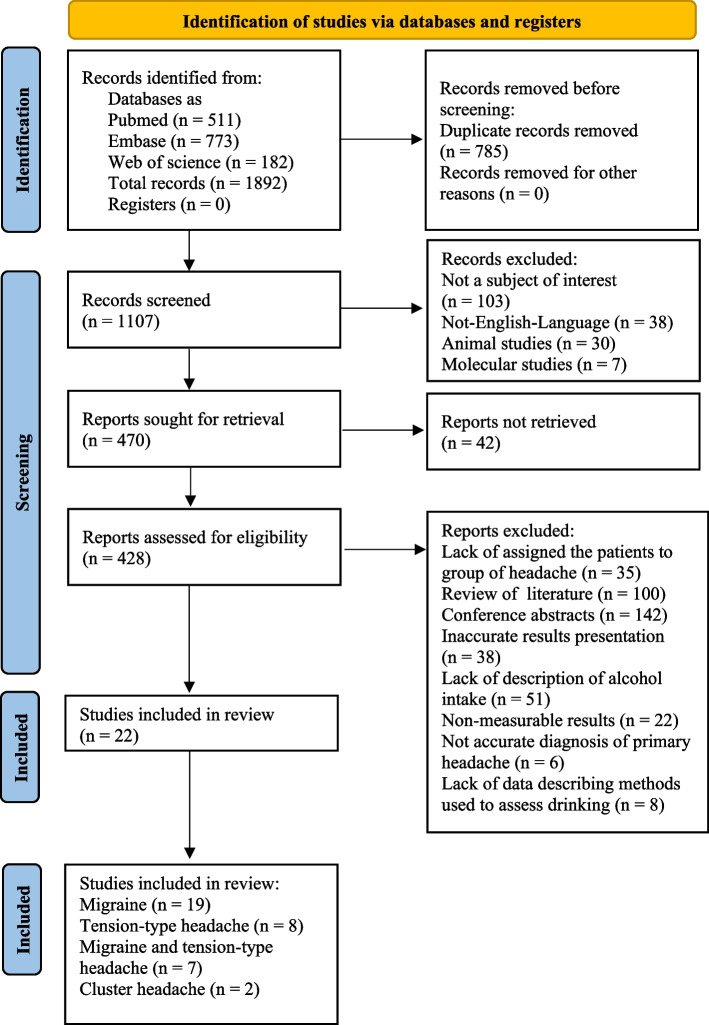
PRISMA 2020 flow diagram

After using the above key terms, 1,892 articles were identified in the three databases. 511 papers were found in PubMed, 773 in Embase and 608 in Web of Science. At the outset, 785 duplicates were excluded. Subsequently, 38 non-English articles, 30 animal studies, seven studies concentrating on molecular pathways to alcohol intake and 562 papers not related to our topic were removed from the remaining records. Then, 142 conference abstracts, 100 reviews, four book chapters and 42 unretrieved studies were not taken into further consideration. Among the full-text articles, 35 had not assigned patients to a specific headache-type or to alcohol intake; 51 studies lacked a description of alcohol intake; 38 papers presented results in an inaccurate way; 22 studies lacked a control group; in 6 articles diagnosis of primary headaches were not based on appropriate criteria and 8 articles do not contain data about methods to define alcohol consumption. Finally, 22 articles [26, 27, 36, 44–62] were retrieved for further analysis. A detailed description of the steps performed during study selection is presented in Fig. 1.

### Study characteristics

The 22 included articles [26, 27, 36, 44–61, 64] came from 10 individual countries, while four papers were international [26, 27, 44, 47]. The majority of these were from Europe: three from Denmark [51, 60, 61], two from Norway [36, 46], four studies conducted in Germany [45, 52, 55, 58] and single papers the Netherlands [59] and Italy [62]. From around the world, one Korean [53], two Turkish [49, 54], one Bangladeshi [50], two United States of America (USA) [48, 57], and one Brazilian [56] were identified.

Most studies were performed with migraine cohorts – these made up 19 out of the 22 papers [26, 27, 36, 45–59, 65]. 11 studies were focused on migraine without any other primary headache [44–50, 53, 56, 58, 59]. All migraine studies [26, 27, 36, 45–59, 65] had a healthy control group without any primary headache. We identified 26 327 migraine participants, including 11 280 alcohol drinkers and 15 047 non-drinkers. The combined control groups represented 99 846 individuals: 59 157 drinkers and 40 689 non-drinkers. In total, all the migraine studies combined included 126 173 participants.

Nine studies [26, 27, 36, 52, 54, 55, 57, 58, 60] analyzed people with tension-type headache (in eight out of the 9 migraine was also evaluated [26, 27, 36, 52, 54, 55, 57, 58]). Only one study was focused only on tension-type headaches [60]. Also, the majority of the studies (eight out of the 9) had a control group of healthy people [26, 27, 36, 52, 54, 55, 57, 58]. In one study [60], migraine was a comparator instead of healthy controls. There were 7937 TTH (2070 drinkers and 5867 non-drinkers). The control group consisted of 13 304 drinkers and 7474 non-drinkers. In total, 28 715 people were included in the 9 TTH studies. We found only two articles on cluster-headache cohorts relevant to our criteria [61, 62]. One study [61] had a control group of healthy people, the second [62] had a non–cluster-headache control group (i.e., migraine). For meta-analysis purposes, there were 391 drinkers and 208 non-drinkers in the cluster-headache category; 292 drinkers and 108 non-drinkers represented the control group.

In 15 studies, headaches were diagnosed based on criteria developed by the International Classification of Headache Disorders (ICHD) [26, 27, 36, 44, 45, 48, 49, 52–55, 57, 58, 61, 62] and its various versions (six out of the 15 employed the latest third edition [26, 27, 44, 48, 49, 53]). Seven articles used International Headache Society (IHS) criteria from 1988 [46, 47, 50, 51, 56, 59, 60].

Criteria to recognize habits for alcohol consumption was various in almost each study. Some of them assessed the drinking by daily alcohol intake [44, 45, 47, 48, 55, 58, 59, 62], part of them measured drinking within one week [44, 47, 51, 53, 60, 61] or month [36, 44, 47, 49, 51]. Additionally, there were cases [56, 57] where division was based on never, current or past drinking. More accurate calculation with amount and various types of alcohol was also conducted in studies [26, 27, 45, 54]. Only few studies [46, 48–50, 52] provided the data about the period in which alcohol drinking was considered and measured. Nineteen studies used questionnaire methods to assess drinking [36, 44–59, 61, 62]. In two cases, questionnaires were supplemented by medical interviews [53, 61]. The rest of the studies were based on information obtained during a medical interview [26, 27, 44].

### Analysis of alcohol consumption

#### Migraine

**Fig. 2:**
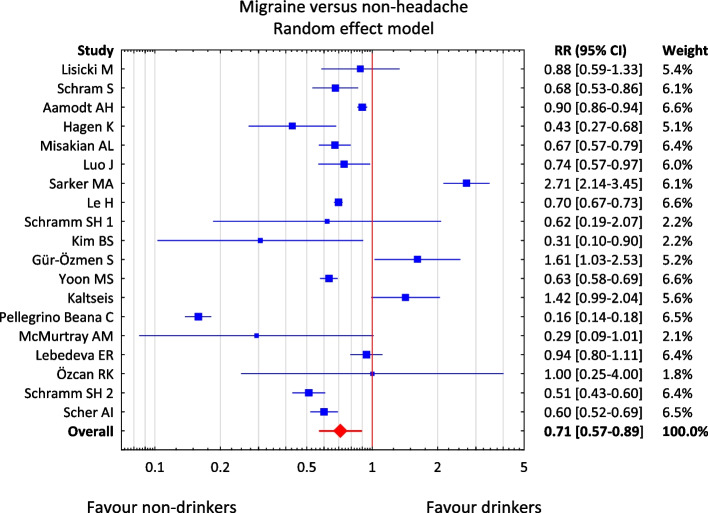
The result of the meta-analysis of the risk of migraine pain in patients who differ in terms of alcohol consumption. Abbreviations: RR – relative risk; CI – confidence interval

The meta-analysis included 19 studies [26, 27, 36, 45–59, 65] presenting data on the presence or absence of migraine pain and assessment of alcohol consumption status (Table 2). Due to the nature of the available data on the status of alcohol consumption, a dichotomous division of the respondents into "non-drinkers" and "drinkers" was used. The total effect obtained in the model is RR = 0.71, and the 95% confidence interval (95% CI) was in the range 0.57–0.89. The results presented in forest graphs (Fig. 2) indicate a significantly lower risk of migraine in people who consume alcohol. In the group of drinkers, the risk of migraine is approximately 1.5 times lower than in the group of non-drinkers (RR = 0.71).

**Table 2:** 19 migraine studies included in the meta-analysis with *p*-value and RR. 11 studies are highlighted in red: *p* is statistically significant (*p* < 0.05). Abbreviations: RR – relative risk; LL – lower limit for RR; UL – upper limit for RR; SE – standard error for RR; *p*-value –– probability value

	**Study**	**Study**	**LL**_**RR**_	**UL**_**RR**_	***p-value***	**Weight**
1	Lisicki M [44]	0,88	0,59	1,33	0,549	5,37%
2	Schram S [45]	0,68	0,53	0,86	0,001	6,14%
3	Aamodt AH [46]	0,90	0,86	0,94	0,000	6,61%
4	Hagen K [36]	0,43	0,27	0,68	0,000	5,12%
5	Misakian AL [47]	0,67	0,57	0,79	0,000	6,39%
6	Luo J [48]	0,74	0,57	0,97	0,031	6,02%
7	Sarker MA [50]	2,71	2,14	3,45	0,000	6,14%
8	Le H [51]	0,70	0,67	0,73	0,000	6,61%
9	Schramm SH 1 [52]	0,62	0,19	2,07	0,436	2,19%
10	Kim BS [53]	0,31	0,10	0,90	0,032	2,51%
11	Gür-Özmen S [54]	1,61	1,03	2,53	0,038	5,16%
12	Yoon MS [55]	0,63	0,58	0,69	0,000	6,56%
13	Kaltseis K [26]	1,42	0,99	2,04	0,055	5,62%
14	Pellegrino Beana C [56]	0,16	0,14	0,18	0,000	6,46%
15	McMurtray AM [57]	0,29	0,09	1,01	0,052	2,11%
16	Lebedeva ER [27]	0,94	0,80	1,11	0,466	6,38%
17	Özcan RK [49]	1,00	0,25	4,00	1,000	1,80%
18	Schramm SH 2 [58]	0,51	0,43	0,60	0,000	6,37%
19	Scher AI [59]	0,60	0,52	0,69	0,000	6,45%
	**Overall**	**0,71**	**0,57**	**0,89**	**0,002**	**100,00%**

When analyzing the data in Table 2 in detail, it is worth noting that in the case of six studies [26, 27, 44, 49, 52, 57], the results are not statistically significant (*p* > 0.05); the results of two studies [50, 54] differ diametrically from the others; and the total result is confirmed by the 11 studies highlighted in red [36, 44–48, 51, 53, 55, 56, 58, 59].

The chi-square test and the I2 statistic were used to assess the non-compliance, i.e., heterogeneity of the studies. The test results—Q = 758, df = 18, *p* < 0.001, T^2^ = 0.186, I^2^ = 97.6%—indicate a significant heterogeneity of the studies included in the meta-analysis. For this reason, the random effect model was used in the meta-analysis. The observed heterogeneity may result from, among others, different status criteria, for example drinker/non-drinker, in individual studies. However, the statistical results did not change when each study was omitted from the sensitivity analysis, indicating that the overall conclusion can be considered reliable.

**Fig. 3:**
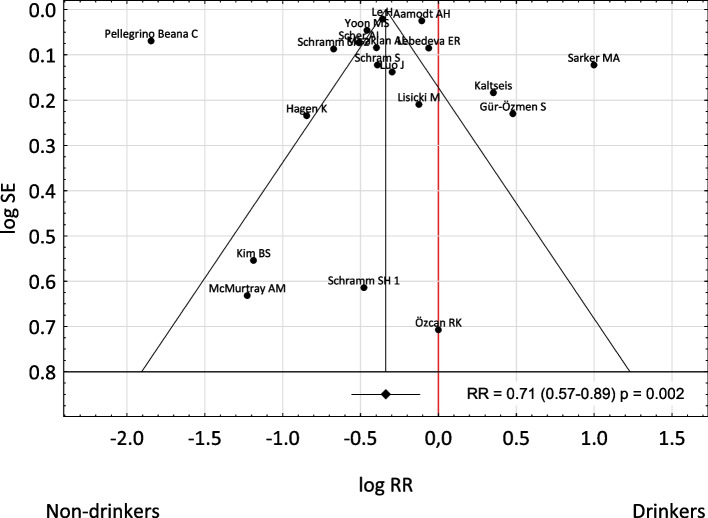
A tunnel graph to assessment the risk of bias of studies in included in meta-analysis. Abbreviations: RR – relative risk; CI – confidence interval

The analysis of the tunnel graph (Fig. 3) and the result of the Trin and Fill procedure, as well as the results of the Begg and Mazumdar test (*p* = 0.243) and the Egger test (*p* = 0.769) indicate the lack of statistically significant publication bias.

#### Tension-type headache

**Fig. 4:**
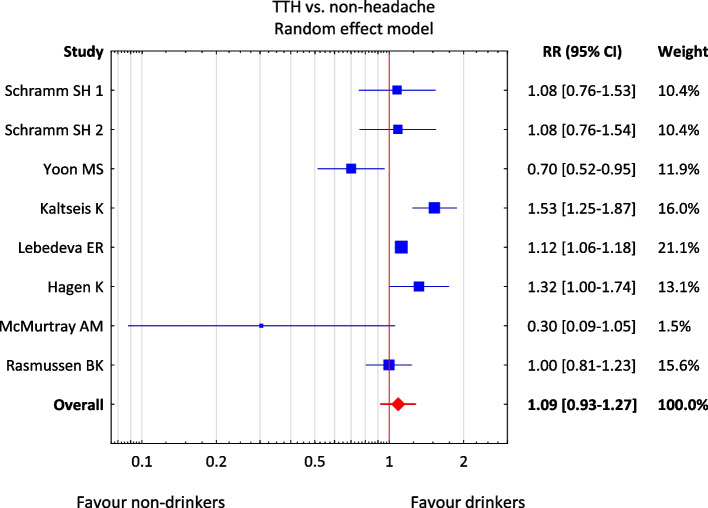
Result of the meta-analysis of the risk of tension headache in patients who differ in alcohol consumption. Abbreviations: RR – relative risk; CI – confidence interval; TTH – tension-type headache

The meta-analysis included 8 out of the 9 studies [26, 27, 36, 52, 55, 57, 58, 60] with data on the incidence of tension-type headache and the assessment of alcohol consumption status, because RR for the study by Gür-Özmen et al. [54] was 0. Due to the nature of the available data on the status of alcohol consumption, a dichotomous division of the respondents into "non-drinkers" and "drinkers" was used. The test results—Q = 24.6, df = 7, *p* = 0.001, T^2^ = 0.030, I^2^ = 71.6%—indicate a significant heterogeneity of the studies included in the meta-analysis. Therefore, a variable effects model was used. In the group of non-drinkers, the risk of migraine attack is higher than in the group of drinkers (RR = 1.09), but the 95% CI (0.93–1.27) contains the value of 1—RR is not significantly different from 1—none of the compared groups differing in alcohol consumption is more exposed to TTH (Fig. 4). The results presented in the form of forest graphs indicate the lack of a statistically significant relationship between the risk of TTH and alcohol consumption. The control groups as a non–tension-type headache in one case [60] do not have any influence on the final results.

#### Cluster headache

The two articles on cluster headaches draw contrasting conclusions. In Lund et al. [61], the risk of cluster headache is significantly lower in non-drinkers (RR = 0.65) while in Lambru et al. [62], the opposite is true: the risk of CH in non-drinkers is higher (RR = 1.54). A synthesis of both papers does not provide any meaningful answer about the relationship between alcohol consumption and cluster headache.

### Risk of bias

Analysis of the 22 included studies revealed 5 cohort studies [36, 45, 52, 55, 58], 11 cross-sectional [26, 27, 44, 46–48, 51, 56, 57, 59, 60] and six case-controls [49, 50, 53, 54, 61, 62]. Of the cohort studies, two [36, 66] received fewer than 8 “yes” answers, therefore according to the assessment criteria from the Methods section above, these were assessed as having moderate risk of bias. The majority of cohort studies were within the range of 3–7 points, thus receiving a high risk of bias [52, 55, 58]. None of the cohort studies had low bias-risk. A detailed description of risk of bias assessment for the cohort studies is presented in Table 3. In cross-sectional studies, six out of the 11 had a high risk of bias [46–48, 51, 56, 60], because they received fewer than 6 positive answers. Three studies were evaluated as moderate risk, with 6 “yes” answers [44, 57, 59]. Two of the remaining cross-sectional papers achieved seven or eight points and therefore were low bias-risk [26, 27]. Table 4 summarizes the assessment of the cross-sectional risk of bias. One of the six case–control studies was assessed as having a high risk of bias [49], two a low risk of bias [50, 62] and three a moderate bias risk [53, 54, 61]. The steps for case–control assessment are presented in Table 5.

**Table 3:** Assessment of risk of bias for cohort studies according to the Joanna Briggs Institute (JBI) checklist

Study authors	Q1	Q2	Q3	Q4	Q5	Q6	Q7	Q8	Q9	Q10	Q11	Overall risk of bias assessment
Schramm S et al. [45]	Yes	Yes	No	Yes	Yes	No	Yes	Yes	Yes	No	Yes	Moderate
Hagen K et al. [36]	Yes	Yes	No	Yes	Yes	No	Yes	Yes	Yes	No	Yes	Moderate
Schramm SH et al. [52]	Yes	Yes	No	Unclear	Yes	No	No	Yes	Yes	No	Yes	High
Yoon MS et al. [55]	Yes	Yes	No	Yes	Yes	No	No	Unclear	Yes	Yes	Yes	High
Schramm SH et al. [58]	Yes	Yes	No	Yes	No	No	Yes	Unclear	Unclear	Unclear	Yes	High

Q1—Were the two groups similar and recruited from the same population?

Q2—Were the exposures measured similarly to assign people to both exposed and unexposed groups?

Q3—Was the exposure measured in a valid and reliable way?

Q4—Were confounding factors identified?

Q5—Were strategies to deal with confounding factors stated?

Q6—Were the groups/participants free of the outcome at the start of the study (or at the moment of exposure)?

Q7—Were the outcomes measured in a valid and reliable way?

Q8—Was the follow-up time reported and sufficiently long for outcomes to occur?

Q9—Was follow-up complete, and if not, were the reasons for this incomplete follow-up described and explored?

Q10—Were strategies to address incomplete follow-up utilized?

Q11—Was appropriate statistical analysis used?

**Table 4:** Assessment of risk of bias for cross-sectional studies according to the Joanna Briggs Institute (JBI) checklist

Study authors	Q1	Q2	Q3	Q4	Q5	Q6	Q7	Q8	Overall risk of bias assessment
Lisicki M et al. [44]	Yes	Yes	No	Yes	No	Yes	Yes	Yes	Moderate
Aamodt AH et al. [46]	Yes	Yes	No	Yes	No	No	Yes	Yes	High
Lebedeva ER et al. [27]	Yes	Yes	Yes	Yes	Yes	Yes	Yes	Yes	Low
Misakian AL et al. [47]	Yes	Yes	No	Yes	No	No	Yes	Yes	High
Le H et al. [51]	No	Unclear	No	Yes	Yes	Yes	Yes	Yes	High
Kaltseis K et al. [26]	Yes	Yes	Yes	Yes	Yes	Unclear	Yes	Yes	Low
Pellegrino Baena C et al. [56]	Yes	Yes	No	Yes	Unclear	No	Yes	Yes	High
McMurtray AM et al. [57]	Yes	Yes	No	Yes	Yes	No	Yes	Yes	Moderate
Rasmussen BK [60]	Yes	Yes	No	Yes	No	No	Yes	Yes	High
Scher AI et al. [71]	Yes	Yes	No	Yes	Yes	No	Yes	Yes	Moderate
Luo J [48]	Yes	Yes	No	No	Yes	Unclear	Yes	Yes	High

Q1—Were the criteria for inclusion in the sample clearly defined?

Q2—Were the study subjects and the setting described in detail?

Q3—Was the exposure measured in a valid and reliable way?

Q4—Were objective, standard criteria used for measurement of the condition?

Q5—Were confounding factors identified?

Q6—Were strategies to deal with confounding factors stated?

Q7—Were the outcomes measured in a valid and reliable way?

Q8—Was appropriate statistical analysis used?

**Table 5:** Assessment of risk of bias for case–control studies according to the Joanna Briggs Institute (JBI) checklist

Study authors	Q1	Q2	Q3	Q4	Q5	Q6	Q7	Q8	Q9	Q10	Overall risk of bias assessment
Lambru G et al. [62]	Yes	Yes	Yes	No	Yes	Yes	Yes	Yes	Yes	Yes	Low
Lund N et al. [61]	Yes	Yes	Yes	No	Yes	Yes	No	Yes	Unclear	Yes	Moderate
Özcan RK et al. [49]	Yes	No	Yes	Yes	Yes	Unclear	Unclear	Yes	No	Yes	High
Sarker MA et al. [50]	Yes	Yes	Unclear	Yes	Yes	Yes	Yes	Yes	Unclear	Yes	Low
Kim BS et al. [53]	Yes	Yes	Yes	No	Yes	Yes	No	Yes	No	Yes	Moderate
Gür-Özmen S et al. [54]	Yes	Unclear	Yes	No	Yes	Yes	Yes	Yes	Unclear	Yes	Moderate

Q1—Were the groups comparable other than the presence of disease in cases or the absence of disease in controls?

Q2—Were cases and controls matched appropriately?

Q3—Were the same criteria used for identification of cases and controls?

Q4—Was exposure measured in a standard, valid and reliable way?

Q5—Was exposure measured in the same way for cases and controls?

Q6—Were confounding factors identified?

Q7—Were strategies to deal with confounding factors stated?

Q8—Were outcomes assessed in a standard, valid and reliable way for cases and controls?

Q9—Was the exposure period of interest long enough to be meaningful?

Q10—Was appropriate statistical analysis used?

## Discussion

The primary objective of our systematic review was to reliably assess alcohol intake in patients suffering from primary headaches, and the secondary objective was to identify a potential answer to the question of whether there is any relationship between alcohol consumption and headache risk. Out of the approximately 1,900 initially selected articles, 22 met the inclusion criteria; however, the majority of these had moderate or high risk of bias. But from our review certain conclusions could be drawn. Alcohol consumption was often considered a trigger or risk factor for migraine or tension-type headache, which was supported by previous studies [31, 67]. The mechanism by which alcohol induces the particular type of headache is unknown [68]. It seems that the theory of the vasodilation of brain vessels after alcohol consumption is insufficient; more probable is pathogenesis involving receptors in the cortex or brainstem [69]. However, some studies did not confirm alcohol influence on primary headaches [51, 70]. The results of our meta-analysis of studies on over 100 000 people indicate a 1.5-lower risk of migraine in people who consume alcohol. To the best of our knowledge, few studies in the literature present similar results—that alcohol decreases the frequency of migraine attacks [38, 46]—but rarely was there any indication of the exact number of potential risks for headache. Despite previous inconclusive results for studies focusing on the relationship between alcohol and headaches, especially in migraine, about one in five headache sufferers believe that alcohol accelerates their particular headache attacks [71]. Due to this stereotype, non-drinking behavior among migraine patients is widespread, which has previously been confirmed [72, 73]. However, our systematic review only considered the simple division of drinkers and non-drinkers, because the majority studies do not distinguish the exact amount of alcohol consumed.

The exact amount of consumed alcohol may have varied effects on headache, e.g., Mostofsky et al. [74] indicate that 1–2 servings of alcohol do not correlate with headache, but five or more servings are associated with increased risk. Therefore, it should be remembered that alcohol consumption may be related to different headache results associated with drinking patterns. Studies show that moderate drinking may reduce the disease burden of mortality in comparison to abstainers [75]. Moreover, low consumption is associated with reduced risk of diabetes and heart attack [76]. However, it is an established fact that heavy drinking leads to serious diseases such as liver cirrhosis, pancreatitis, dementia and malignant neoplasms [77]. In addition, these results may differ between among different age, gender and work-status cohorts [78]. However, due to methodological issues, our meta-analysis could not consider these confounders. We also were not able to recalculate consumption descriptors to countable units. Even if some studies provide such estimations, the amount is different in each article. Additionally, there is no standardized alcohol assessment method in these publications. The included studies used units, grams, glasses, drinks, pints and milliliters, which makes it impossible to recalculate to a unified amount.

Whereas the World Health Organization (WHO) states that there is no safe alcohol dose [19], Panconesi et al. conclude that low consumption is not a contraindication for headache patients [79]. However, each patient makes individual decisions based on their own experience. Headache after a certain amount of alcohol is likely to induce behavioral reactions (i.e., alcohol-intake adjustment). Similarly, common beliefs may influence patients habits, e.g., the conviction that “red wine causes migraine”, even if studies present conflicting evidence [80, 81]. Consequently, it seems likely that people with migraine to some extent avoid alcohol, which would be one interpretation of our results. For this reason, people with migraine may gain unforeseen healthcare benefits, e.g., avoiding negative effects of alcohol consumption such as gastrointestinal cancers [82], which can be partially confirmed by Elser et al. [83].

A second explanation for the results presented in our meta-analysis might encompass a certain protective role of alcohol with regards to migraine. However, according to this idea, populations with higher migraine prevalence should have lower alcohol consumption. For example, due to religious requirements, people in Iran consume considerably less alcohol than Europeans [22, 84]; nevertheless, migraine prevalence in Iran is 15.1% [85] while in Europe it is 35% [86]. In Europe, alcohol consumption is higher than in Asian countries, but in Europe alcohol as a trigger is reported more frequently than it is in Asia [87]. Therefore, this hypothesis seems a less likely explanation for our results.

According to our results, the relationship between alcohol and headache is more pronounced in migraine than in tension-type headache [32, 88]. However, it is worth noting that more studies concentrate on migraine than TTH (19 of the included studies vs 9); moreover, the prevalence of TTH is greater than that of migraine [89]. The result from our meta-analysis was that there is a lack of a relationship between the risk of TTH and alcohol consumption. Similar results are also reported in the literature [36, 60]. Again, there are also studies where alcohol is reported as a TTH trigger [27]. Similar to our migraine meta-analysis, some confounders could not be considered, e.g., quantity and type of alcohol, gender, and episodic/chronic form of TTH. In the literature, cluster headaches are associated with alcohol and often with nitrates [81]. However, data about this topic is also inconclusive [80]. Unfortunately, the studies included in our analysis did not allow unequivocal answers in this area. Only two articles satisfied the inclusion criteria, and in Lund et al. [61] cluster headache is significantly less prevalent in non-drinkers, but in the second study—Lambru et al. [62]—this risk is higher in non-drinkers.

Assessment of alcohol consumption is challenging, because the results are dependent on the patient's honesty. Patients sometimes have a tendency not to admit their drinking habits [90]. It has been proved that self-reported alcohol consumption by patients can be underestimated; therefore, more reliable methods such as toxicological hair analysis may help to provide stronger evidence [91]. Of the studies included in our analysis, 19 were based only on questionnaires while five included interviews with patients. However, these limitations are to some extent discounted by the number of studies included and the cultural diversity of participants.

This study has some limitations. First of all, the existing studies present data in a heterogeneous way, which may have led to inaccurate results, and do not provide an exhaustive array of information. Information on the gender of participants was unavailable for analysis. So, the question of who is drinking more with a primary headache is still to be addressed. Additionally, only a few of the studies divided participants into migraine with and without aura. Therefore, there was insufficient data to analyze the relationship between alcohol and aura, and the data that does exist is inconsistent [65, 86]. As mentioned in the discussion above, alcohol consumption assessment is strongly based on patients’ honesty. If there is misleading data in questionnaires or during medical interviews, their overall subsequent analysis is also distorted. Therefore, this meta-analysis was not able to assess particular variables of alcohol in primary headaches, e.g., gender, division into type of migraine, TTH, cluster headache or type of alcohol drinking, which could be key to various previously reported results. The relatively low number of cluster-headache studies also does not allow an assessment of any correlation with alcohol drinking. Moreover, some of the studies included in our review do not present results in an accurate way or do so without assigning patients to specific headaches. Therefore, it was not possible for our meta-analysis to contain all those studies where drinking was described with primary headache. The ways describing alcohol consumption habits were variously presented in almost each study, therefore could develop the observed heterogeneity among migraine analysis. Also, the majority of the studies had high or moderate risk of bias. Thus, our results should be interpreted with care.

## Conclusions

Alcohol consumption and migraine are inversely correlated. The exact mechanism behind this observation may indicate that migraine leads to alcohol-avoidance rather than alcohol having a protective role against migraine. There is no relationship between TTH and drinking. However, there is a need to conduct further studies related to primary headaches and alcohol consumption with low risk of bias. Additionally, patients and physicians should consider the latest medical knowledge to avoid perpetuating the myths about alcohol consumption and primary headaches. Additionally, it would be useful to check whether migraine patients enjoy the advantages or disadvantages of less drinking.

## Key points

The meta-analysis showed a 1.5-lower risk of migraine in people who consume alcohol. However, migraine patients consume less alcohol for various reasons. Consequently, migraine patients can avoid the negative effects of alcohol consumption but also positive aspects of drinking such as protection from heart attack or diabetes, a sociable life or they may deny themselves possibilities for enjoyment. Therefore, patients with primary headache need to determine for themselves the association between alcohol and headache without any myths and influences. The results of our meta-analysis are that there is a lack of a relationship between the risk of TTH and alcohol consumption. Further studies should present exact levels of alcohol intake in standardized units, clearly state the division of migraine, TTH and cluster headache into subtypes, distinguish drinkers and non-drinkers in terms of gender and include the type of alcohol. More cluster headache studies should be conducted.

## Data Availability

The datasets generated and/or analysed during the current study are available from the corresponding author on reasonable request.
